# Inhibition of HEWL fibril formation by taxifolin: Mechanism of action

**DOI:** 10.1371/journal.pone.0187841

**Published:** 2017-11-13

**Authors:** Mohsen Mahdavimehr, Ali Akbar Meratan, Maryam Ghobeh, Atiyeh Ghasemi, Ali Akbar Saboury, Mohsen Nemat-Gorgani

**Affiliations:** 1 Department of Biological Sciences, Institute for Advanced Studies in Basic Sciences (IASBS), Zanjan, Iran; 2 Department of Biology, Science and Research Branch, Islamic Azad University, Tehran, Iran; 3 Institute of Biochemistry and Biophysics, University of Tehran, Tehran, Iran; 4 Stanford Genome Technology Center, Stanford University, Palo Alto, California, United States of America; University of Pittsburgh School of Medicine, UNITED STATES

## Abstract

Among therapeutic approaches for amyloid-related diseases, attention has recently turned to the use of natural products as effective anti-aggregation compounds. Although a wealth of *in vitro* and *in vivo* evidence indicates some common inhibitory activity of these compounds, they don’t generally suggest the same mechanism of action. Here, we show that taxifolin, a ubiquitous bioactive constituent of foods and herbs, inhibits formation of HEWL amyloid fibrils and their related toxicity by causing formation of very large globular, chain-like aggregates. A range of amyloid-specific techniques were employed to characterize this process. We found that taxifolin exerts its effect by binding to HEWL prefibrillar species, rather than by stabilizing the molecule in its native-like state. Furthermore, it’s binding results in diverting the amyloid pathway toward formation of very large globular, chain-like aggregates with low β-sheet content and reduced solvent-exposed hydrophobic patches. ThT fluorescence measurements show that the binding capacity of taxifolin is significantly reduced, upon generation of large protofibrillar aggregates at the end of growth phase. We believe these results may help design promising inhibitors of protein aggregation for amyloid-related diseases.

## Introduction

Protein misfolding and its subsequent deposition in different organs and tissues may cause serious degenerative diseases collectively known as amyloidoses, which are characterized by the presence of long-unbranched amyloid fibrils [1]. Until recently, more than 25 human peptides and proteins have been identified to form pathological amyloid aggregates [2]. Despite few similarities in their amino acid sequences and tertiary structures, they can form amyloid fibrils with common features in morphology and biochemical properties [3,4]. The unfolding and assembly of these structures appear to be initiated by destabilization of their native conformation, leading to formation of partially unfolded intermediates [5–8]. Based on these observations, inhibition of amyloid formation and/or clearance of fibrillar structures may provide an effective therapeutic approach for treatment of amyloid-related diseases [5, 9–11]. Among various strategies developed to reduce amyloid aggregation, a simple and practical approach is the use of synthesized or natural small molecules as aggregation suppressors, both under *in vitro* and *in vivo* conditions. To this end, naturally-occurring polyphenols have been found to be one of the most effective inhibitors [12,13], with their presence in daily foods [14], making them attractive therapeutic agents.

Hen egg white lysozyme (HEWL) is commonly used as a model protein to study the mechanism of amyloid fibril formation and inhibition by small molecules [15, 16]. A variety of polyphenols, including (−)-epicatechin gallate [17], myricetin [18], curcumin and kaempferol[19] have been found effective. Additionally, we have recently reported that two naturally-occurring polyphenols, namely rosmarinic acid and resveratrol have the capacity to inhibit HEWL amyloid fibril formation, disaggregate preformed fibrils, and attenuate their related cytotoxicity [20]. Taxifolin (also known as dihydroquercetin) is a flavonoid found in grapes, citrus fruits, onions, green tea, olive oil, and several herbs (such as milk thistle) [21]. Besides its antitumor, hepatoprotective, and anti-inflammatory activities [22], it is a potent antioxidant, which may contribute to its cardiovascular and neuroprotective properties [23]. As for amyloid-related diseases, some investigators have found that taxifolin could be used as a novel inhibitor of Aβ42 aggregation [24,25]. Moreover, Saito *et al*. have reported that taxifolin prevents amyloid-β oligomer assembly and fully sustains cognitive and cerebrovascular function in cerebral amyloid angiopathy model mice [26]. However, the exact mechanism by which this natural antioxidant modulates the protein aggregation process has not been clearly understood. Therefore, in the present study, the effect of taxifolin on fibrillation of HEWL was evaluated. Our results clearly indicate that taxifolin effectively inhibits HEWL amyloid fibrillation and their related toxicity via directing the HEWL aggregation process toward formation of very large globular, chain-like aggregates. Moreover, its optimal binding to protein and the residues involved were identified by performing fluorescence anisotropy and molecular docking.

## Materials and methods

### Material

HEWL (EC 3.2.1.17), Thioflavin T (ThT), Nile red, Congo red, and taxifolin were purchased from Sigma (St. Louis, MO, USA). All other chemicals were obtained from Merck (Darmstadt, Germany) and were reagent grade.

### Sample preparation

Protein concentration was determined spectrophotometrically at 280 nm, using an extinction coefficient (ɛ^1mg/ml^) of 2.63 at 280 nm [27]. Stock solution of taxifolin was prepared at 50 mM, using dimethysulfoxide (DMSO) as solvent, and was stored at -20°C until use. The final concentration of DMSO did not exceed from 0.2% in the incubating solutions containing the highest concentration of taxifolin.

### HEWL amyloid fibril formation

HEWL amyloid fibrils were prepared as previously reported with some modifications [28]. Briefly, the protein was dissolved in glycine buffer 50 mM (pH 2.2) to a final concentration of 50 μM, and aliquots were incubated at 57°C (without or with taxifolin) while being stirred at 500 rpm to induce amyloid fibril formation. The molar ratios of taxifolin to protein used in this study were 0.5:1, 1:1, and 2:1.

### Fluorescence measurements

#### Thioflavin T fluorescence assay

All fluorescence experiments were carried out on a Cary Eclipse VARIAN fluorescence spectrophotometer. Formation of HEWL fibrils was monitored by following the increase in ThT fluorescence intensity using a mixture of 2 μM protein solutions and 10 μM ThT, with excitation fixed at 440 nm and emission at 482 nm. Excitation and emission slit widths were set at 5 and 10 nm, respectively. The acquired data from ThT fluorescence measurements were fitted to the sigmoid curve, depicted by the following equation [29]:

$$F=Fmin+(\frac{Fmax}{{1+e}^{-[\frac{\left(t-t0\right)}{\tau }]}})$$

where F is the fluorescence intensity at time t, F_min_ and F_max_ represent fluorescence intensity at initial time and saturation phase of incubation, respectively. t is the incubation time and t_0_ is the time required to obtain 50% of maximal fluorescence. The value of τ was obtained by nonlinear regression. Apparent growth rate constant (k_app_) and lag phase time were determined to be 1/τ and t_0_−2τ, respectively. In all experiments, ThT fluorescence measurement was done in triplicate and the mean of the three measurements was determined.

#### Nile red fluorescence assay

For Nile red fluorescence measurements, aliquots of the HEWL solutions incubated with various concentrations of taxifolin were removed at different time intervals and diluted to a final concentration of 2.5 μM in glycine buffer (50 mM, pH 2.2) containing 10 μM Nile red. Samples were excited at 530 nm and emission spectra were recorded from 540–800 nm, with 5 and 10 nm slit widths for excitation and emission, respectively. Nile red fluorescence experiments were performed in triplicate.

#### Tryptophan fluorescence assay

For tryptophan fluorescence measurements, aliquots of the HEWL solutions incubated without or with various concentrations of taxifolin for 7 days were removed and diluted to a final concentration of 2 μM in glycine buffer (50 mM, pH 2.2). Samples were excited at 295 nm, and the spectrum was recorded between 300 and 400 nm, with 5 nm slit width for both excitation and emission.

#### Fluorescence anisotropy measurements

Nile red fluorescence anisotropy measurements were monitored at an excitation wavelength of 555 nm at room temperature. The fluorescence emission (λ_max_ = 641 nm) was recovered through a GG455 filter (Oriel) to remove the excitation light scattering. For these experiments, the labeled protein (50 μM) was titrated without or with various concentrations of taxifolin (25, 50, and 100 μM). The steady-state anisotropy (A) is given by [30]:

$$A=\frac{I_{VV}{-G\times I}_{VH}}{I_{VV}{+\;G\times 2I}_{VH}}$$

where I_VV_ and I_VH_ are the intensities measured with vertically polarized excitation, as indicated by the first subscript, and detected through vertically or horizontally oriented emission polarizers, respectively, as indicated by the second subscript. As the light is not equally transmitted through both parallel and perpendicular oriented polarizers, a correction was performed. The correction factor, named G factor, is measured using horizontally polarized excitation and is given by the following expression [30]:

$$G=\frac{I_{HV}}{I_{HH}}$$

### Congo red binding assay

50 μl of the HEWL solutions previously incubated at various concentrations of taxifolin for 7 days was added to 950 μl of a Congo red solution (20 μM in 5 mM potassium phosphate and 0.15 M sodium chloride, pH 7.4). After 8 h of incubation at room temperature, absorbance spectra were recorded between 400–600 nm. The amount of bound Congo red was quantified by CRB (M) = (A_540_/25295)—(A_488_/46306), where CRB (M) is the molar concentration of bound Congo red, and 25295 and 46306 are the molar extinction coefficients of bound and unbound Congo red, respectively [31].

### Far-UV CD measurement

CD spectra were recorded using an AVIV 215 spectropolarimeter (Aviv Associates, Lakewood, NJ, USA) and a 0.05 mm path cell. Aliquots of the HEWL solutions incubated at various concentrations of taxifolin were removed after 7 days and diluted (to final concentration of 15 μM) in glycine buffer (50 mM, pH 2.2), and the spectra were recorded in the range of 190–260 nm. The percent of alpha helix, beta sheet, turns and unordered structures were determined for each sample using the SELCON program on the online server DICHROWEB [32].

### SDS-PAGE analysis

Non-reducing SDS polyacrylamide gel electrophoresis (SDS-PAGE) was performed. Aliquots (15μl) corresponding to 7-day-old HEWL incubated under amyloidogenic conditions, either alone or in the presence of various concentrations of taxifolin, were taken and mixed with 5μl of sample buffer and applied onto the gel (12%). Electrophoresis was performed at constant voltage at 100 V. Gel was stained with 0.025% (w/v)Coomassie brilliant blue followed by multiple destaining.

### Atomic force microscopy

Aliquot of HEWL samples incubated at different concentrations of taxifolin for 7 days were removed and diluted 25 fold with deionized water. Then, 10 μl of diluted sample was placed on freshly cleaved mica and dried at room temperature. Images were acquired in non-contact mode using a quantitative Atomic Force Microscopy (ARA-AFM, Ara-Research Company, Iran). Images were processed using Imager (version 1.01, Ara-Research Company). The diameter of 5 particles was measured randomly and the average and standard deviations were then calculated.

### Dynamic light scattering

All measurements were carried out using a zeta potential and particle size analyzer (Brookhaven Instrument, Holtsville, NY11742-1896, USA). For size distribution measurements, aliquots of samples incubated without or with various concentrations of taxifolin, at a final concentration of 5μM, were filtered through a 0.2 μm syringe filter followed by illumination by a laser of 657 nm with a fixed detector angle of 90° at RT. DLS experiments were performed at least in triplicate.

### MTT assay

Human neuroblastoma SH-SY5Y cells, obtained from Pasteur Institute (Tehran, Iran), were cultured in DMEM-F12 medium, supplemented with 10% fetal bovine serum, streptomycin (100 μg/ml) and penicillin (100U/ml), and kept at 37°C in a 5% CO_2_ humidified atmosphere. Growth medium was changed three times a week. Cells were seeded in 96-well plate at a density of 2 × 10^4^ cells/well, and the medium was changed before incubation with HEWL amyloid aggregates. For cytotoxicity experiments, protein samples taken from solutions incubated with various concentrations of taxifolin (0–100 μM) under amyloidogenic conditions were added to the cells (in a final concentration of 10 μM) and left for 24 h. Cells treated with 50 mM glycine buffer (pH 2.2) was used as control. Then, 10 μl of MTT stock solutions (5 mg/ml in PBS) were added to 100 μl of DMEM-F12 containing 10% fetal bovine serum, followed by incubation for 3 h. Solutions were aspirated, and cells were treated with DMSO for 15 min, followed by absorbance reading at 570 nm on an ELISA reader (Expert 96, Asys Hitch, Ec Austria). Results were expressed as percentage of MTT reduction relative to the control cells, assuming that the absorbance of the control cells was 100%. All measurements were made in triplicates.

### Molecular docking study

For molecular docking calculations, the crystal structure of the HEWL was obtained from the Protein Data Bank (PDB ID: 3WUN) and the structure of the ligand taxifolin was obtained from zinc database (ZINC ID:105082). Prior to docking, all water molecules were removed from the PDB file. Using Auto Dock tools 1.5.6 Program [33], all hydrogen atoms were also added to the protein, and Kollman and Gasteiger charges were used for HEWL and taxifolin, respectively. Throughout the dockings, the protein molecule was set to be rigid while the ligand molecule was considered to be flexible, and one active bond of the ligand was set rotatable. The grid volume was arranged big enough to cover the entire surface of the protein. A total of 100 runs were performed to more accurately find the most appropriate binding site holding the lowest binding energy (calculated using the Autodock scoring function). After verifying the residues lining this binding site (Asp52, Gln57, Ile58, Asn59, Trp62, Trp63, Ile98, Asp101, and Trp108), they were set flexible for another 250 runs of molecular dockings to further define the role of the protein residues involved in the interaction with ligand. Finally, the ligand pose with the lowest binding energy score was selected as the best binding mode to HEWL. The secondary-structure images were created using VMD 1.9.3 [34].

## Result and discussion

In recent years, various synthetic and naturally-occurring compounds have been introduced as potent inhibitors of amyloid fibril formation[5,9–11,35,36]. However, the majority of these molecules may not serve as effective therapeutic leads due to their toxicity and/or poor blood-brain barrier permeability. In the present study, we investigated the anti-amyloidogenic effects of taxifolin, a naturally occurring polyphenols commonly present in the human diet, with no detectable toxicity [21,26,37]. To determine if taxifolin may influence HEWL fibril formation, various concentrations of this compound were added to the incubation medium. The amyloid fibrillation process was then investigated by employing a number of techniques. Our results demonstrated that taxifolin effectively inhibited HEWL fibril formation and its related cytotoxicity in a concentration-dependent manner. To further decipher its mechanism of action in hindering HEWL fibrillogenesis, a range of amyloid-specific techniques were utilized. Finally, fluorescence anisotropy and molecular docking were performed to further characterize the process.

### Effect of taxifolin on HEWL amyloid fibril formation

**Fig 1:**
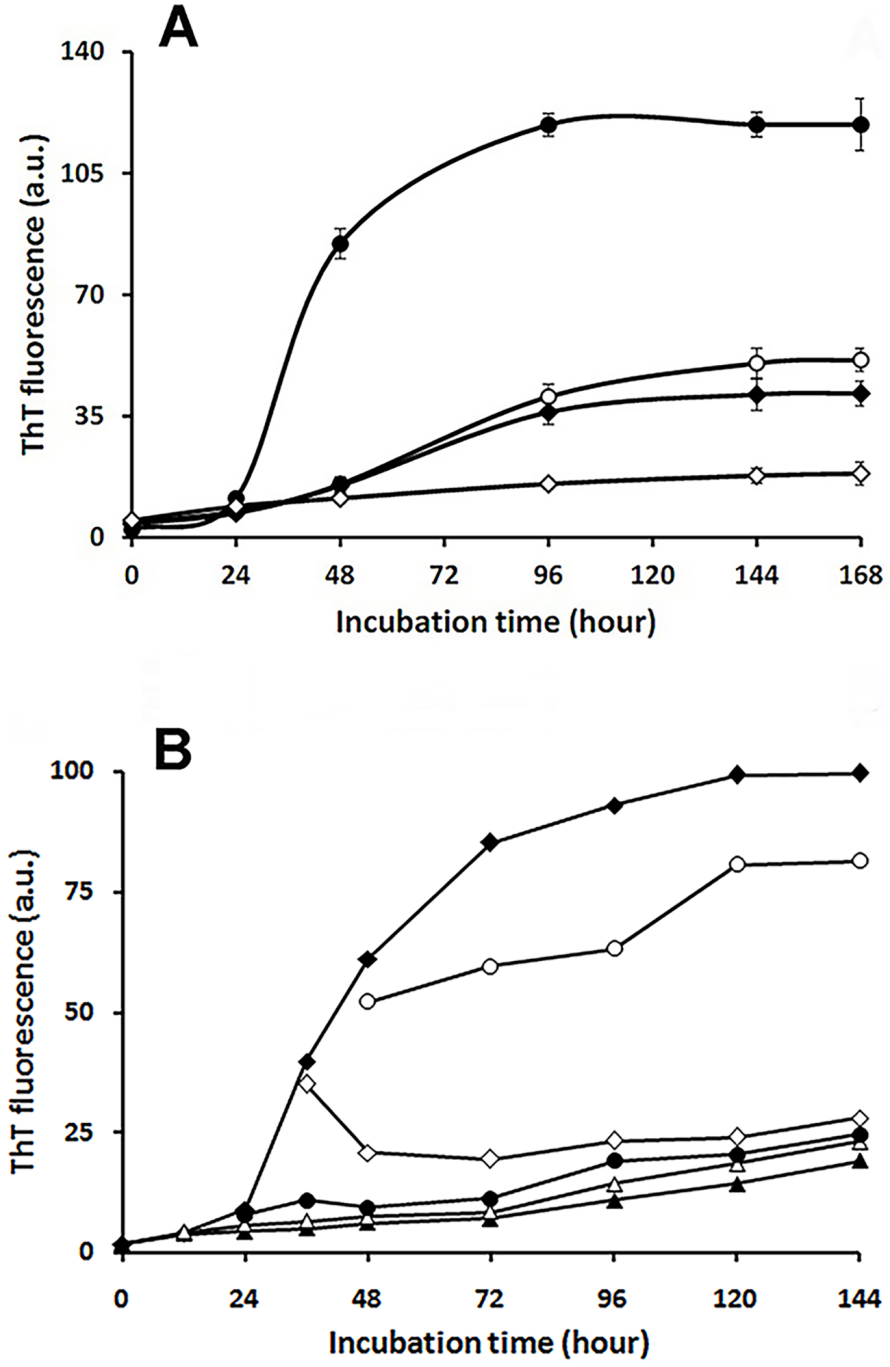
Effects of taxifolin on the kinetics of HEWL amyloid fibrillation as monitored by ThT fluorescence assay. (A) Protein samples (50 μM) were incubated in 50 mM glycine buffer (pH 2.2) at 57°C either alone (●) or with 25 (○), 50 (♦), or 100 (◊) μM taxifolin. Each experiment was performed in triplicate. (B) Taxifolin (at a final concentration of 100 μM) was added immediately (▲), or after regular time intervals of 12 (△), 24 (●), 36 (◊), and 48 (○) hours, followed by ThT fluorescence measurements. HEWL incubated alone (♦) was also indicated as control.

ThT fluorescence assay was carried out to monitor growth of HEWL amyloid fibrils and to examine the effect of taxifolin on the rate of fibril formation. Kinetics of HEWL amyloid formation in the absence and presence of taxifolin (25, 50, and 100 μM) are shown in Fig 1A, indicating a concentration-dependent decrease in ThT fluorescence. In accord with our previous reports [20,28], HEWL displayed a nucleated polymerization mechanism, in agreement with the nucleation dependent polymerization of other amyloidogenic peptides and proteins [38–40].

Meanwhile, presence of higher concentrations of taxifolin (up to 200 μM), did not lead to any significant reduction in ThT fluorescence intensity (Figure A in S1 File). The ThT fluorescence kinetics analyses indicated that the lag time of HEWL fibrillation was slightly increased at 25 and 50 μM (Table 1).

**Table 1:** Effect of taxifolin on the kinetics parameters of HEWL fibrillization determined by ThT fluorescence assay.

	K_app_ (h^-1^)	Lag time (h)	Amplitude (a.u.)
**0 μM taxifolin**	3.32 ± 0.22	27.25 ± 1.78	119.00 ± 7.81
**25 μM taxifolin**	1.14 ± 0.07	28.04 ± 1.86	51.20 ± 3.41
**50 μM taxifolin**	1.28 ± 0.13	27.93 ± 2.84	41.53 ± 4.22
**100 μM taxifolin**	0.55 ± 0.10	-^a^	18.42 ± 3.54

^a^The lag time could not be determined in the presence of 100 μM taxifolin since aggregation was not observed.

For samples incubated with 100 μM of taxifolin, the lag time could not be determined since aggregation was significantly hindered (Fig 1A and Table 1). A dose-dependent reduction in the final amplitude and apparent growth rate constant (k_app_) of the fibrillation curves were observed (Table 1). These results indicated that taxifolin effectively suppressed HEWL fibril formation without significant changes on the onset of the fibrillation process, suggesting that it has the capacity to interact with prefibrillar species.

To test this hypothesis, the protein was placed under amyloidogenic conditions, and taxifolin (at a final concentration of 100 μM) was added either immediately or after regular time intervals of 12, 24, 36, and 48 hours (corresponding to the middle of lag time, and beginning, middle, and end of the growth phase, respectively), followed by ThT fluorescence measurements up to 6 days. As depicted in Fig 1B, incubation with taxifolin led to a significant reduction in fluorescence intensity. However, its inhibitory effect was considerably reduced when added after 48 h of incubation. Based on this observation, it is suggested that taxifolin exerts its inhibitory effects by binding to species produced at the early stages of HEWL aggregation, which is in accord with earlier reports on studies involving a number of peptides and proteins [13, 41–43]. However, in the course of growth of amyloidogenic species and generation of large protofibrillar aggregates, the binding capacity of taxifolin was significantly reduced (Fig 1B).

**Fig 2:**
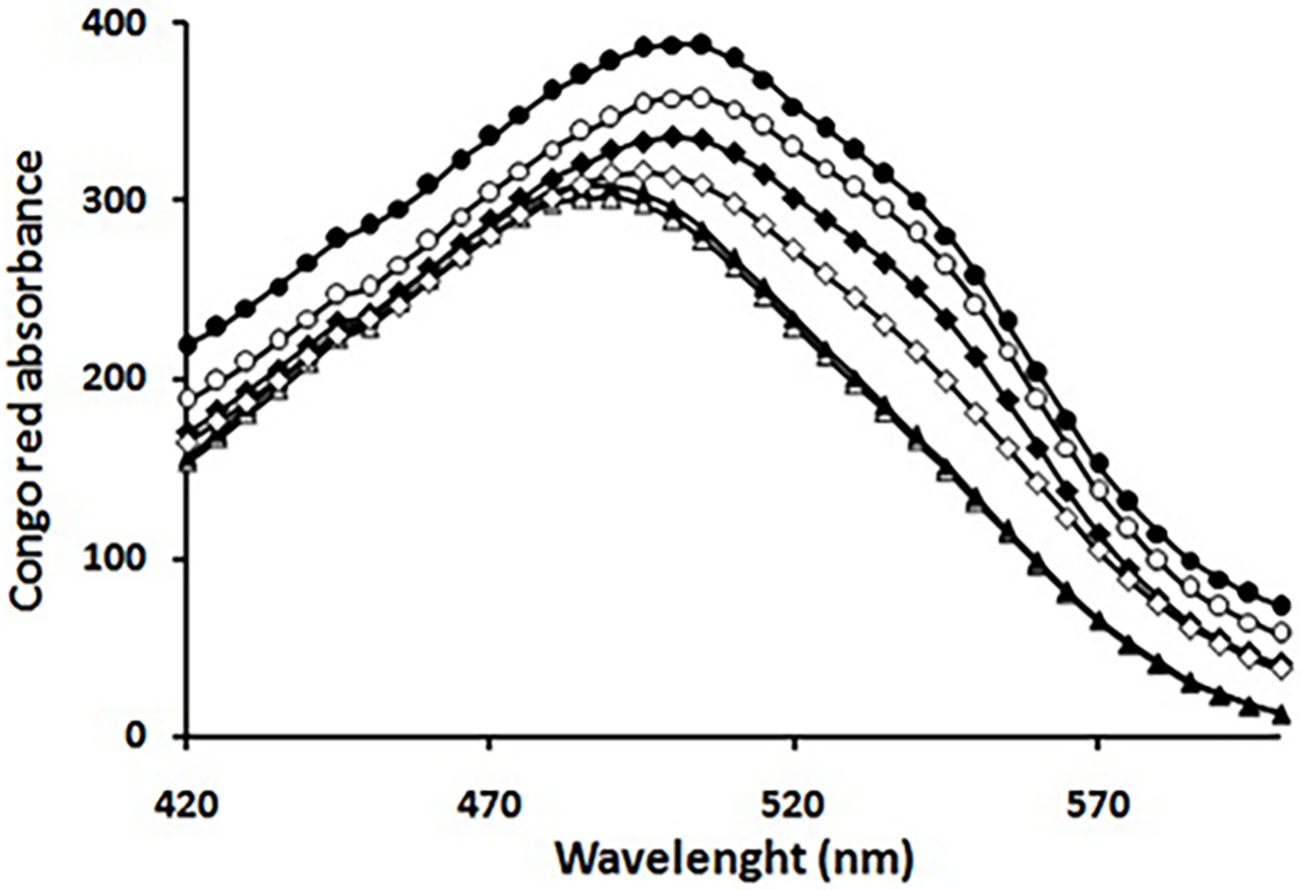
Congo red binding absorption spectra of HEWL in the absence and presence of taxifolin. Protein samples (50 μM) were incubated in 50 mM glycine (pH 2.2) at 57°C either alone (●) or with 25 (○), 50 (♦), or 100 (◊) μM taxifolin for 7 days. Congo red absorbance alone and in the presence of HEWL monomer are indicated by (△) and (▲), respectively.

Congo red binding assay, as a complementary evaluation of amyloid fibril formation, was performed to probe the presence of β-sheet structures associated with amyloid fibrils. As shown in Fig 2, a marked enhancement in Congo red absorbance accompanied with a red shift was observed upon 7 days of incubation. Moreover, a second shoulder peak at around 540 nm, indicative of a strong binding affinity between Congo red and HEWL, was also observed, signifying the presence of a substantial amount of amyloid fibrils [44]. However, this was effectively prevented by taxifolin in a concentration dependent manner (Fig 2 and Table A in S1 File).

**Fig 3:**
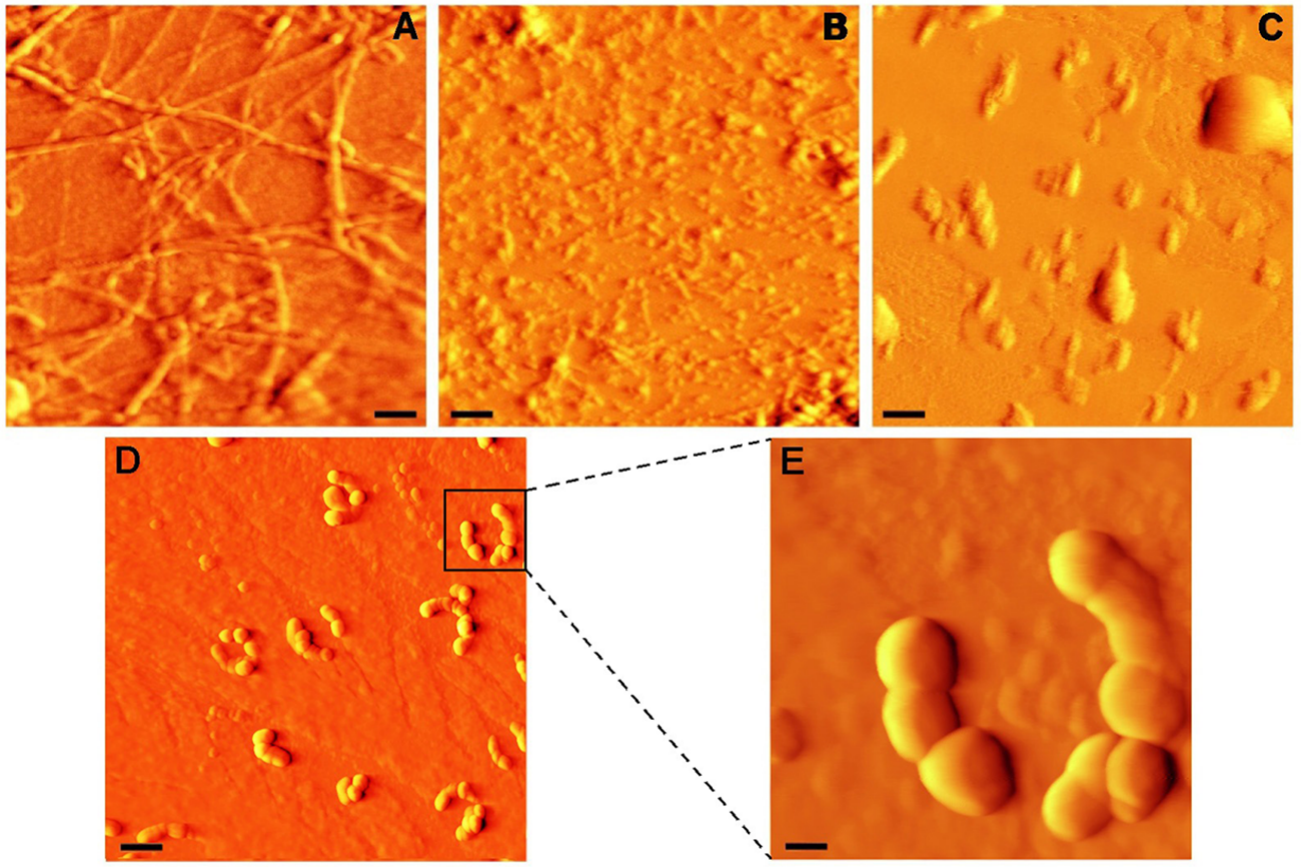
AFM images of HEWL incubated without or with taxifolin. Protein samples (50 μM) were incubated in 50 mM glycine (pH 2.2) at 57°C either alone (A) or with different concentrations of 25 (B), 50 (C), and 100 (D) μM taxifolin for 7 days. (E) An enlarged view of (D). The scale bars represent 500 nm (A-C), 2000 nm (D), and 400 nm (E).

Fig 3 shows AFM images of HEWL, incubated for 7 days under amyloidogenic conditions. As illustrated in Fig 3A, in the absence of taxifolin, well-defined mature fibrils with typical amyloid morphology were formed. Interestingly, we observed various types of amyloid fibrils in control samples, including straight, worm-like and rope-like fibrils, and annular structures (Fig 3A and Figure B in S1 File). On the other hand, in protein samples incubated with 25 μM taxifolin, formation of such fibrillar structures was prominently inhibited, with the appearance of small protofibrillar structures (Fig 3B). In the presence of 50 μM taxifolin, even these structures disappeared, and instead, amorphous aggregates with various diameters (up to 1000 nm) were observed (Fig 3C). In samples containing 100 μM taxifolin, very large globular, chain-like aggregates with diameters of approximately 500–1000 nm (with an average diameter of 765 ± 47.2 nm)were seen (Fig 3D and 3E). Similar to this observation, large chain-like assemblies were observed in the bis(heptyl)-cognitin-treated Aβ samples [45]. This was a further confirmation that in the presence of taxifolin, formation of mature fibrillar structures was strongly inhibited.

**Fig 4:**
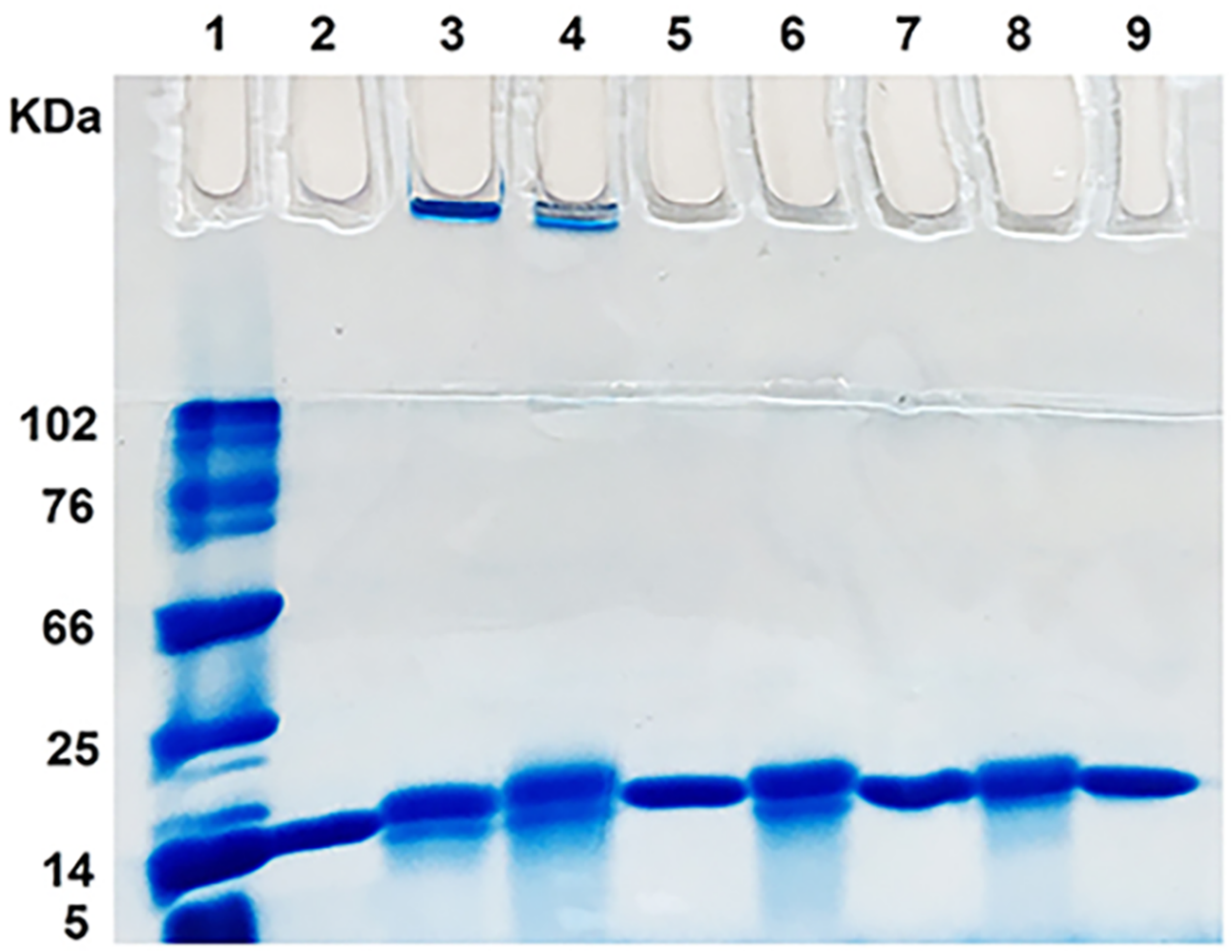
SDS-PAGE analysis of HEWL samples alone or with various concentrations of taxifolin at day 0 (lanes 2, 5, 7, and 9) and day 7 (lanes 3, 4, 6, and 8). The lanes are: lane 1: protein marker; lanes 2 and 3: HEWL alone; lanes 4 and 5: HEWL with 25 μM taxifolin; lanes 6 and 7: HEWL with 50 μM taxifolin; lanes 8 and 9: HEWL with 100 μM taxifolin. SDS-PAGE analyses were performed under non-reducing conditions.

Finally, SDS-PAGE and DLS experiments were employed to explore size distribution of HEWL species. Aliquots of the samples without or with taxifolin were withdrawn at 0 and 7 days of incubation and subjected to SDS-PAGE. As shown in Fig 4, the protein incubated alone displayed both high molecular weight assemblies (corresponding to amyloid fibrils) trapped in the well of the stacking gel and a range of low-molecular weight bands corresponding to acid-induced hydrolysis of the monomeric structure[46,47]. A similar pattern was observed for samples containing 25 and 50 μM taxifolin, indicating that the polyphenol did not protect the protein against acidic proteolysis at these concentrations. However, the high molecular weight assemblies were not detected in samples containing 50 μM taxifolin and at 100 μM concentration, only the band corresponding to native HEWL appeared in the gel (Fig 4). Interestingly, no band corresponding to the large aggregates produced in the presence of 100 μM taxifolin (Fig 3D and 3E) was observed, presumably due to disaggregation of these assemblies by SDS (data not shown), similar to those reported for other proteins [48].

**Fig 5:**
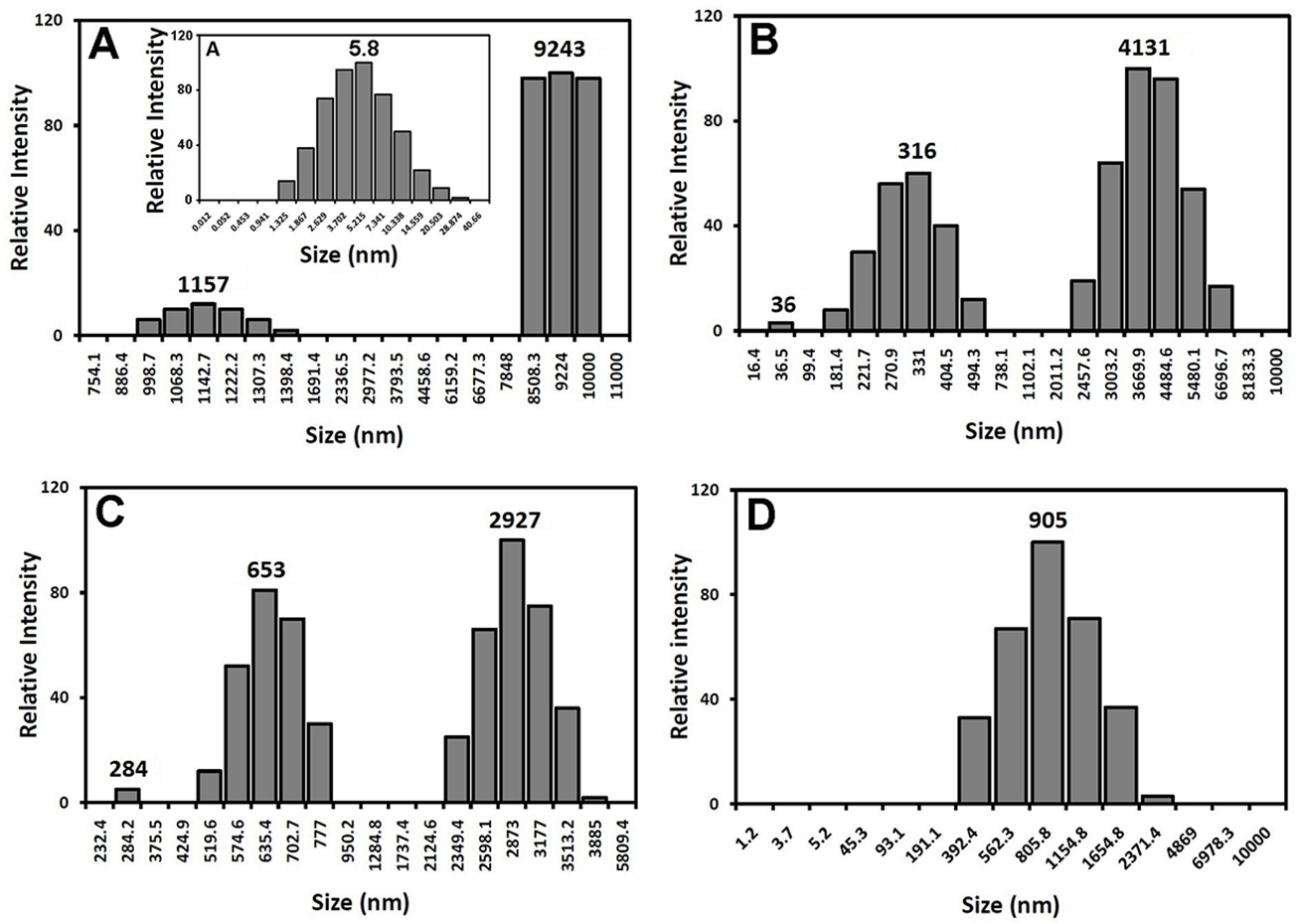
Size distribution of HEWL incubated without or with taxifolin. Protein samples, incubated with various concentrations of taxifolin for 7 days, were diluted to a final concentration of 5 μM followed by DLS measurements. Data presented in the inset (A) indicates the size of HEWL monomer.

Fig 5 shows a typical distribution of the hydrodynamic radii of protein samples incubated with various concentrations of taxifolin. As indicated in Fig 5A, after 7 days incubation under amyloidogenic conditions, control samples formed typical amyloid fibrils, showing an average diameters of 1157 and 9243 nm, indicative of formation of prefibrillar and mature fibrils, respectively. On the other hand, a dose-dependent decrease in diameter was observed in samples containing increasing amounts of taxifolin (Fig 5B–5D), suggesting inhibition of amyloid fibril formation. We suggest that globular morphology of species produced in the presence of 100 μM taxifolin may be account for smaller hydrodynamic radius of these aggregates (Fig 5D).

### Effects of taxifolin on structural/conformational changes of HEWL

**Fig 6:**
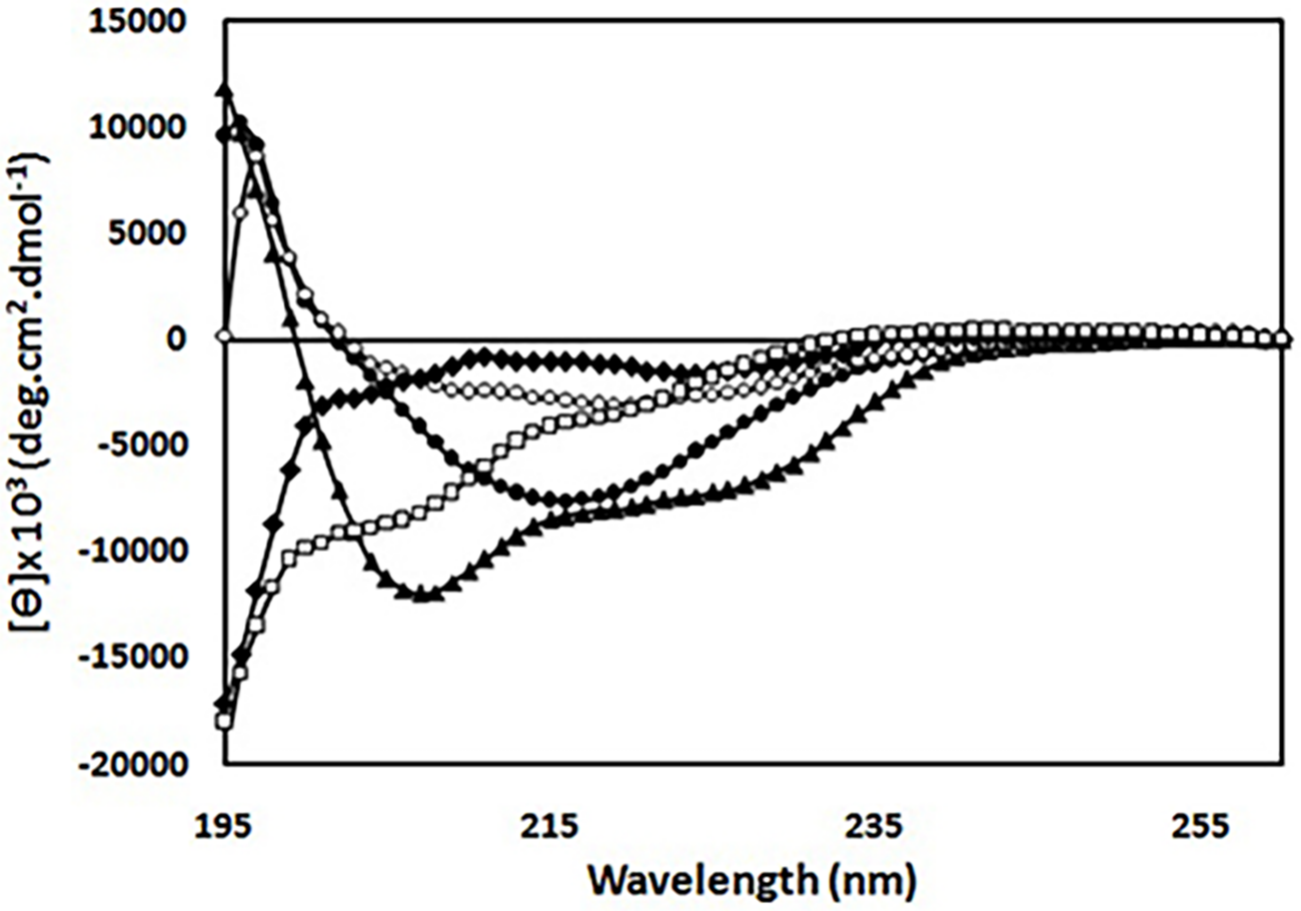
Far-UV CD spectra of HEWL in the absence and presence of taxifolin. Protein samples (50 μM) were incubated in 50 mM glycine (pH 2.2) at 57°C, either alone (●) or with 25 (○), 50 (♦), or 100 (□) μM taxifolin for 7 days. CD spectrum of HEWL monomer is indicated by (▲).

To investigate whether structural/conformational changes of HEWL brought about by the amyloidogenic conditions (acidic pH and high temperature) could be protected by taxifolin, far-UV CD spectra, Nile red and tryptophan fluorescence studies were carried out. As indicated in Fig 6, far-UV CD spectrum of native HEWL was changed from a predominant α-helical to a β-sheet-rich structure, characterized by a major negative peak at around 216 nm. None of the taxifolin concentrations afforded complete protection, although the characteristic change of the spectrum-namely the appearance of a large negative peak around 216 nm-was significantly hindered in the presence of taxifolin (Fig 6).

A summary of the secondary structure contents of HEWL have also been provided in Table 2. While all taxifolin-containing incubations showed a decrease in β-sheet content, in the case of 50 and 100 μM taxifolin, a significant reduction in α-helical content and a concomitant increase in unordered fraction was also observed. These results indicated that HEWL structural changes from native to a partially-unfolded structure have not been inhibited by taxifolin (Table 2).

**Table 2:** The secondary structure contents of HEWL incubated alone or with various concentrations of taxifolin for 7 days under amyloidogenic conditions.

	α-helix	β-sheet	Turn	Unordered
**Fresh HEWL**	35	18	23	24
**0 μM taxifolin**	15	35	29	21
**25 μM taxifolin**	16	27	24	33
**50 μM taxifolin**	6	21	27	46
**100 μM taxifolin**	7	18	31	44

**Fig 7:**
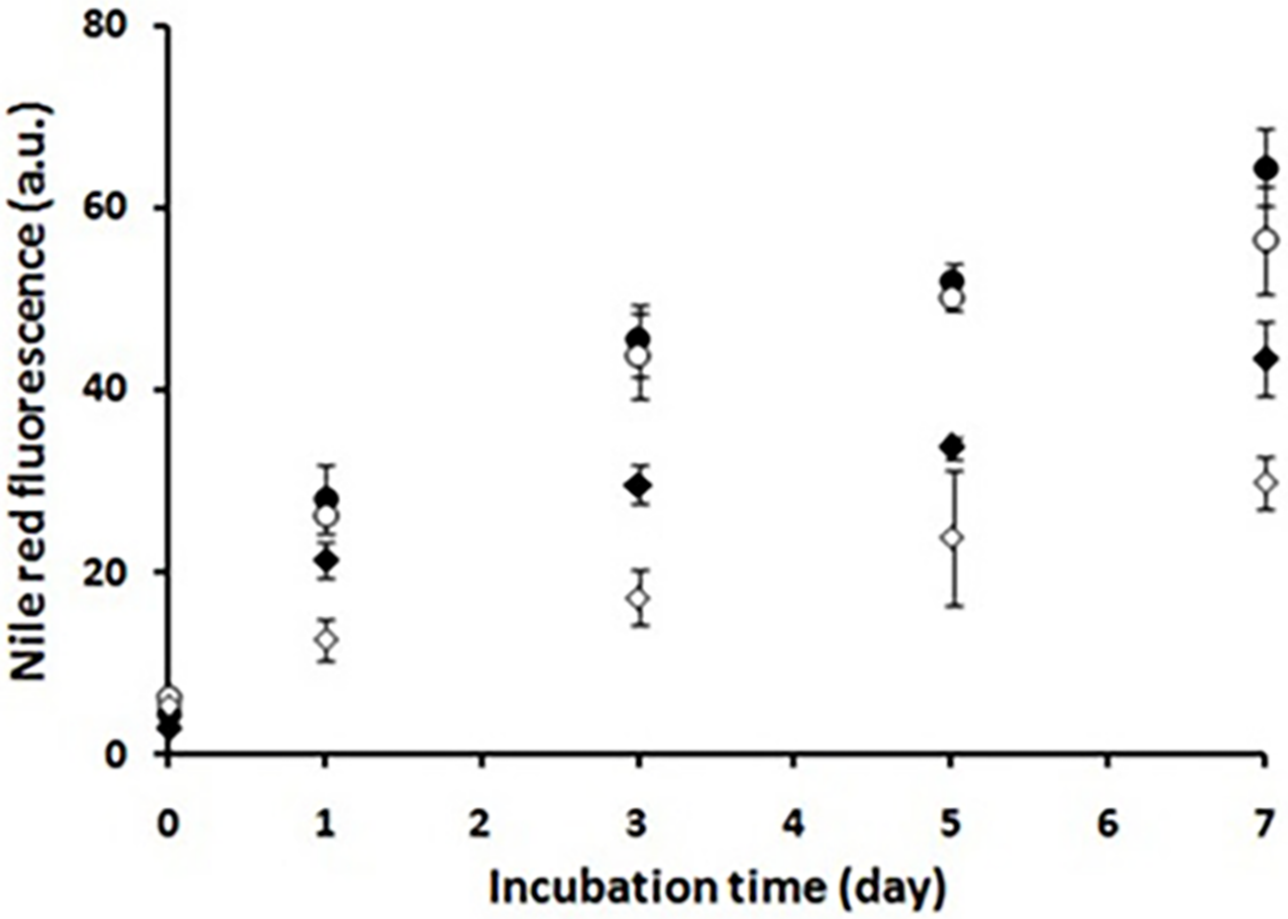
Effect of taxifolin on surface hydrophobicity of HEWL. Protein samples (50 μM) were incubated in 50 mM glycine (pH 2.2) at 57°C either alone (●) or with 25 (○), 50 (♦), or 100 (◊) μM taxifolin and Nile red fluorescence intensity was measured after regular time intervals up to 7 days. Each experiment was performed in triplicate.

It is well-established that incubation of HEWL under amyloidogenic conditions induces conformational changes, characterized by exposure of hydrophobic regions on the surface of protein [28,49]. For samples incubated with taxifolin, a pronounced dose-dependent decrease in Nile red fluorescence intensities was observed which was accompanied by a shift to longer wavelengths (Fig 7 and Figure C in S1 File). This finding may be explained by the ability of taxifolin to bind hydrophobic surfaces, as observed for other polyphenols [50,51]. It appears therefore that blocking hydrophobic patches may be one of the mechanisms by which taxifolin inhibits HEWL amyloid fibrillation, in accordance with previous reports [13,20,52,53].

**Fig 8:**
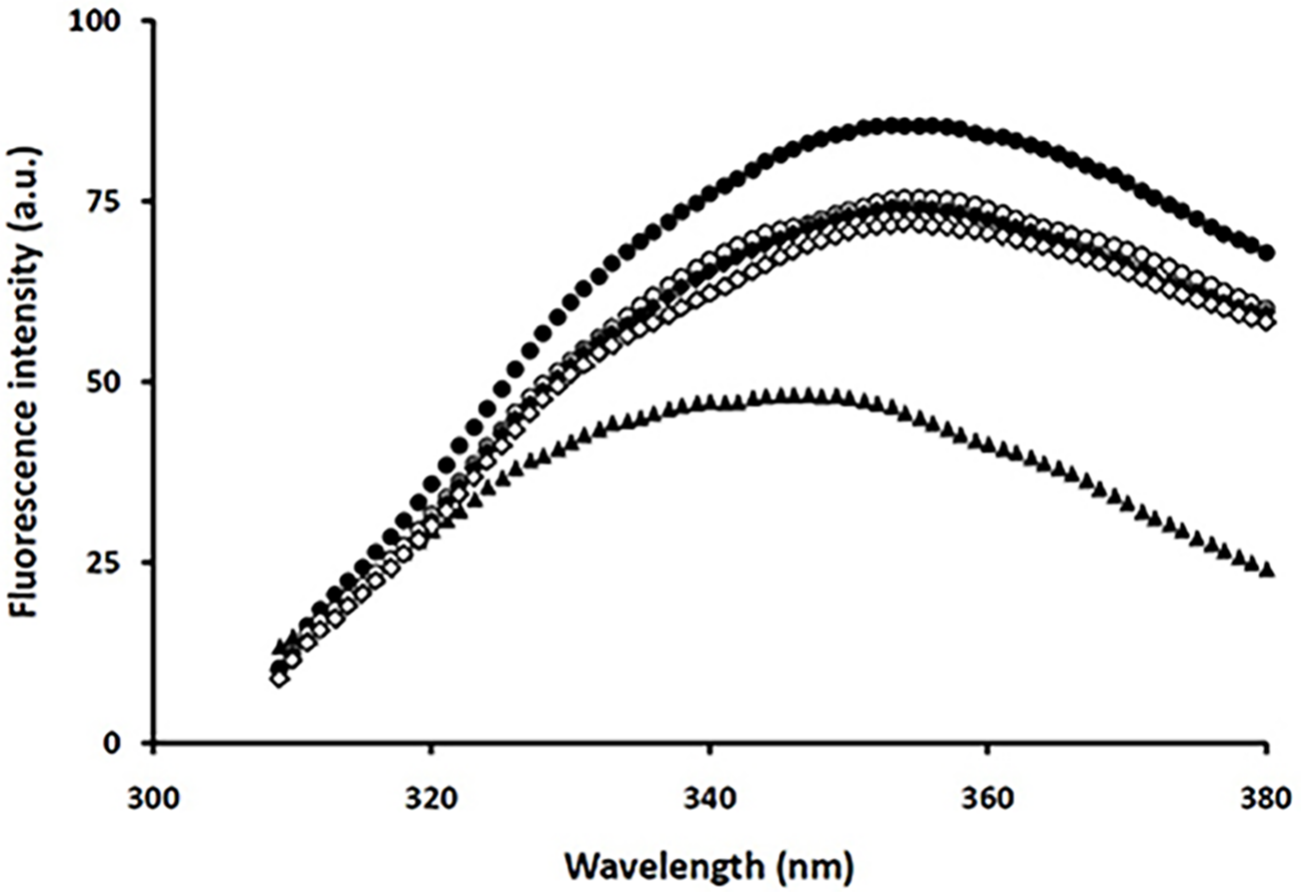
Effect of taxifolin on the tertiary structural changes of HEWL. Protein samples (50 μM) were incubated in 50 mM glycine (pH 2.2) at 57°C either alone (●) or with 25 (○), 50 (♦), or 100 (◊) μM taxifolin for 7 days, followed by tryptophan fluorescence intensity measurements. Tryptophan fluorescence of HEWL monomer is indicated by (▲).

For a better interpretation of our observations, tryptophan fluorescence, which is more sensitive to tertiary structural changes, was measured. As shown in Fig 8, a red-shift in fluorescence emission with an increase in its intensity, indicative of a conformational change in the polar environment of the protein and a partial loss of its native tertiary structure, was observed in control samples. Although the presence of taxifolin somewhat reduced the intensity of tryptophan fluorescence emission, none of the concentrations employed inhibited the transition to longer wavelengths (Fig 8), suggesting that HEWL structural changes were not inhibited, in accord with our far-UV CD data (Fig 6). Taken together, these observations suggest that taxifolin displays its inhibitory effects by redirecting the HEWL aggregation pathway toward formation of very large globular aggregates with low β-sheet content and reduced solvent-exposed hydrophobic patches.

### Characterization of HEWL-taxifolin interaction

Fluorescence anisotropy, and molecular docking analyses were utilized to gain further insights into interaction of taxifolin with HEWL.

Fluorescence anisotropy was used to confirm the interaction between taxifolin and Nile red-labeled HEWL. The fluorescence anisotropy value of Nile red-labeled HEWL in the absence of taxifolin was 0.3305 ± 0.0345 (Table 3). Upon addition of taxifolin a significant decrease in the measured anisotropy was observed (Table 3), suggesting interaction of taxifolin with HEWL. This decrease maybe due to a distinct conformation of protein, induced by taxifolin, in which energy transfer occur between two or more probes, leading to a lower anisotropy for the acceptor probe [54].

**Table 3:** Anisotropy of Nile red-labeled HEWL before and after addition of various concentrations of taxifolin.

	Measured anisotropy
**HEWL**	0.3305 ± 0.0345
**HEWL-25 μM taxifolin**	0.1586 ± 0.0030
**HEWL-50 μM taxifolin**	0.1610 ± 0.0070
**HEWL-100 μM taxifolin**	0.1760 ± 0.0030

**Fig 9:**
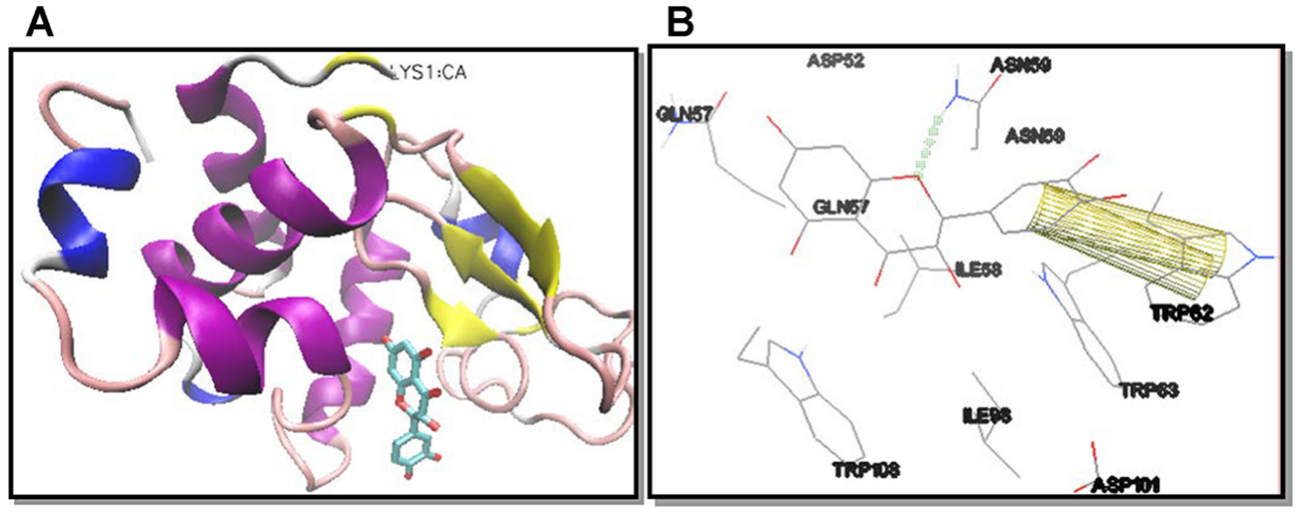
Binding mode of taxifolin to HEWL. (A) The active site of HEWL as the optimal binding site for taxifolin. Taxifolin is depicted in cyan in stick model. Protein backbone of HEWL is shown in cartoon model. The N-terminal of HEWL is displayed on top (LYS1). The secondary structure of HEWL is depicted as follows: β-strand: yellow; α-helix: purple; 3/10 helix: blue; random coil: white; turn: pink. (B) HEWL residues surrounding taxifolin are shown with numbers. Hydrogen bond within 1 Å is represented as dotted green line formed between taxifolin and Asn59. The π-π interaction is shown as yellow tubes between taxifolin and Trp62.

In order to further characterize the interaction between HEWL and taxifolin, molecular docking was performed. The best binding energy score of -7.69 kcal mol^-1^ obtained from docking runs corresponded to a putative binding site located in the cleft, between the α and β domains, which has shown to lie the HEWL active site [55] (Fig 9A). As shown in Fig 9B, this binding site was surrounded by six residues of HEWL β-domain (Asp52, Gln57, Ile58, Asn59, Trp62 and Trp63) and by three residues of HEWL α-domain (Ile98, Asp101 and Trp108). The β-domain of HEWL has been shown as the aggregation-prone region of the protein [46, 56]. More specifically, according to Tokunaga *et al*. residues 54−62 in the β-domain of HEWL act as the aggregation core for amyloid fibril formation [57]. In this core segment, taxifolin has formed a hydrogen bond with Asn59 while its phenyl ring has established π-π interaction with the indole ring of Trp62 (Fig 9B). Curcumin and kaempferol have also been suggested to have similar π-π interactions with this residue [19]. The fact that taxifolin has displayed a binding to the aggregation-prone HEWL β-domain has also been reported in previous studies investigating other polyphenols, including myricetin [18], curcumin [19, 58], kaempferol [19], quercetin, and resveratrol [59]. Interestingly, the interaction with Asn59 and Trp62 in the aggregation-prone β-domain of HEWL could explain the conformational changes detected by anisotropy measurement(Table 3).

### Cytotoxicity of HEWL aggregates

**Fig 10:**
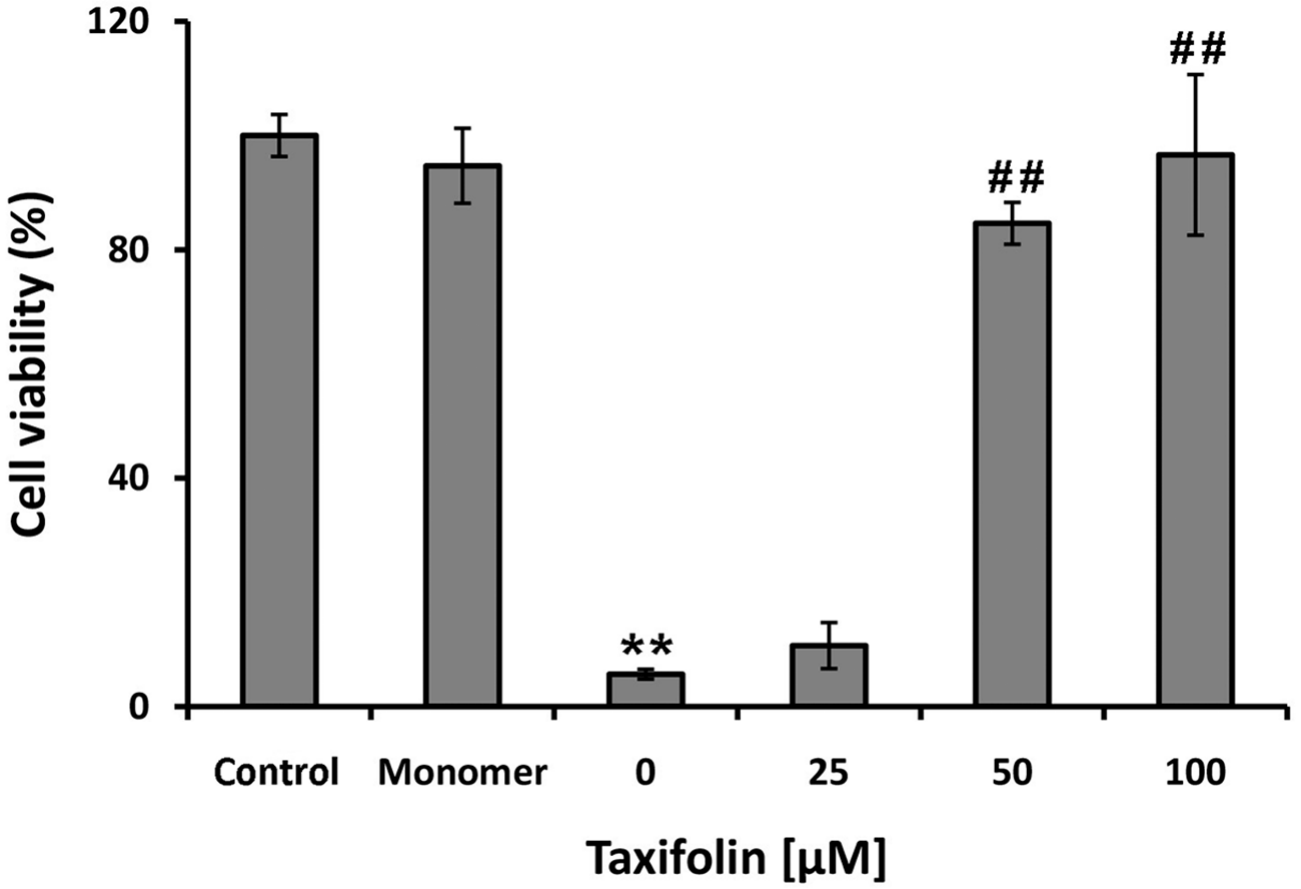
Cytotoxicity of HEWL aggregates produced in the absence and presence of taxifolin. SH-SY5Y cells were treated with HEWL fibrils aged alone or in the presence various concentrations of taxifolin for 24 h. The data were expressed as percentage of values in untreated control cells and each value represents the mean ± SD(n = 3). **p<0.005, significantly different from control cells.^##^p<0.005, significantly different from cells exposed only to HEWL fibrils.

To examine toxicity of aggregates produced in the presence of taxifolin, MTT assay was performed on SH-SY5Y cells exposed to protein samples incubated with increasing amounts (0–100 μM) of taxifolin for 7 days. While no cytotoxicity was detected for HEWL monomer, cell viability decreased significantly after a 24h exposure to HEWL amyloid fibrils (Fig 10). For samples incubated with 25 μM taxifolin also a marked cytotoxicity was found, suggesting toxicity of small protofibrillar structures (Figs 3B and 10). However, in the present of 50 and 100 μM taxifolin, the cell viability was rescued to 85 ± 3.66 and 97 ± 14.05 (Fig 10), respectively, indicating that related aggregates are significantly nontoxic. As exposure of hydrophobic patches in the course of protein aggregation is a crucial and common feature of misfolded toxic species [28,60], we suggest that the presence of taxifolin inhibits the exposure of hydrophobic regions, as indicated by Nile red fluorescence measurements (Fig 7), leading to formation of non-toxic aggregate species.

### Mechanism of HEWL fibrillation inhibition by taxifolin

There are many reports demonstrating that polyphenols are effective inhibitors of protein fibrillation [12,13,17–20], through interaction with one or more of the amyloidogenic species, produced during the course of the aggregation process. For instance, some polyphenols prevent amyloid formation by interacting with and stabilizing native structure of proteins [47,58,61]; while others bind to prefibrillar structures and redirect amyloidogenic polypeptides into unstructured, off-pathways oligomers [62], or toward an alternative non-toxic disordered (amorphous) aggregation pathway [20]. Recently, Hirohata *et al*. showed they may exert their anti-amyloidogenic effects through binding to monomer, oligomer and fibrillar structures of Aβ [63]. For taxifolin, our flexible docking results demonstrated a specific binding site in a HEWL cleft, as outlined above. Fluorescence anisotropy measurement also indicated the interaction between taxifolin and Nile red-labeled HEWL (Table 3), which was in accord with the docking results. Based on these findings, it is reasonable to propose that taxifolin may exert its inhibitory effects through preferential binding to and stabilizing the native state of HEWL, thereby protecting the protein against conformational changes. However, results obtained by far-UV CD and tryptophan fluorescence measurements demonstrated that both secondary and tertiary structural changes of HEWL brought about by the amyloidogenic conditions (acidic pH and high temperature) were not inhibited in the presence of taxifolin, even in samples containing 100μM polyphenol (Figs 6 and 8, and Table 2). Taking these findings, it seems quite unlikely that protecting the native protein from structural changes to be taxifolin’s mechanism of action. One may therefore ask what is the mechanism by which taxifolin inhibits HEWL amyloid fibril formation?

Since an increase in the total protein concentration led to a decrease in the inhibitory effect of taxifolin (Figure D in S1 File), we suggest that a step involving an association reaction is affected[64]. Formation of very large globular, chain-like aggregates (Fig 3D and 3E) explicitly rules out the possibility of the HEWL fibrillation pathway being redirected into formation of off-pathway oligomers or amorphous aggregates, as the mechanism of action of taxifolin. On the other hand, the fact that the lag phase of fibril formation was not affected by the presence of taxifolin (Fig 1A and Table 1) suggests that it may inhibit amyloid formation by acting on species produced after this stage of the fibrillation process. Interestingly, when the polyphenol was added after regular time intervals of incubation, we observed a significant decrease in the ThT fluorescence intensity (Fig 1B), making this proposition likely. However, HEWL-binding features of taxifolin were largely reduced concomitant with the generation of large protofibrillar aggregates (Fig 1B), presumably due to decrement of structural flexibility in the course of amyloid fibril formation [28]. Thus, it appears that inhibition of HEWL amyloid fibrillation occurs by binding of taxifolin to prefibrillar species that lie directly in the course of fibril formation. We suggest this binding of taxifolin to hydrophobic surfaces, exposed in the course of the fibrillation process, shifts the equilibrium in the aggregation pathway by promoting the formation of very large globular aggregates, with low content of surface-exposed hydrophobic regions (Figs 3D, 3E and 7). It remains unclear how taxifolin promotes conversion of HEWL prefibrillar species into very large globular, chain-like aggregates, instead of formation of off-pathway conformers, or large amorphous aggregates, a mechanism displayed by some other polyphenols [20,47,58,60–62]. As the protein bears a net positive charge under acidic condition used for amyloid fibrillation, a probable reason for generation of such chain-like assemblies is the existence of repulsive forces between the aggregate species that cause the aggregates to find the configuration in which repulsive forces are minimized, i.e., the arrangement of aggregates in a linear chain. Thus, small molecules, including polyphenols, may exert their inhibitory effects on various stages of fibrillations, and with different mechanisms. Such diversity of action may lead to the formation of aggregate species with very distinct conformations, morphologies and toxic properties.

## Conclusion

**Fig 11:**
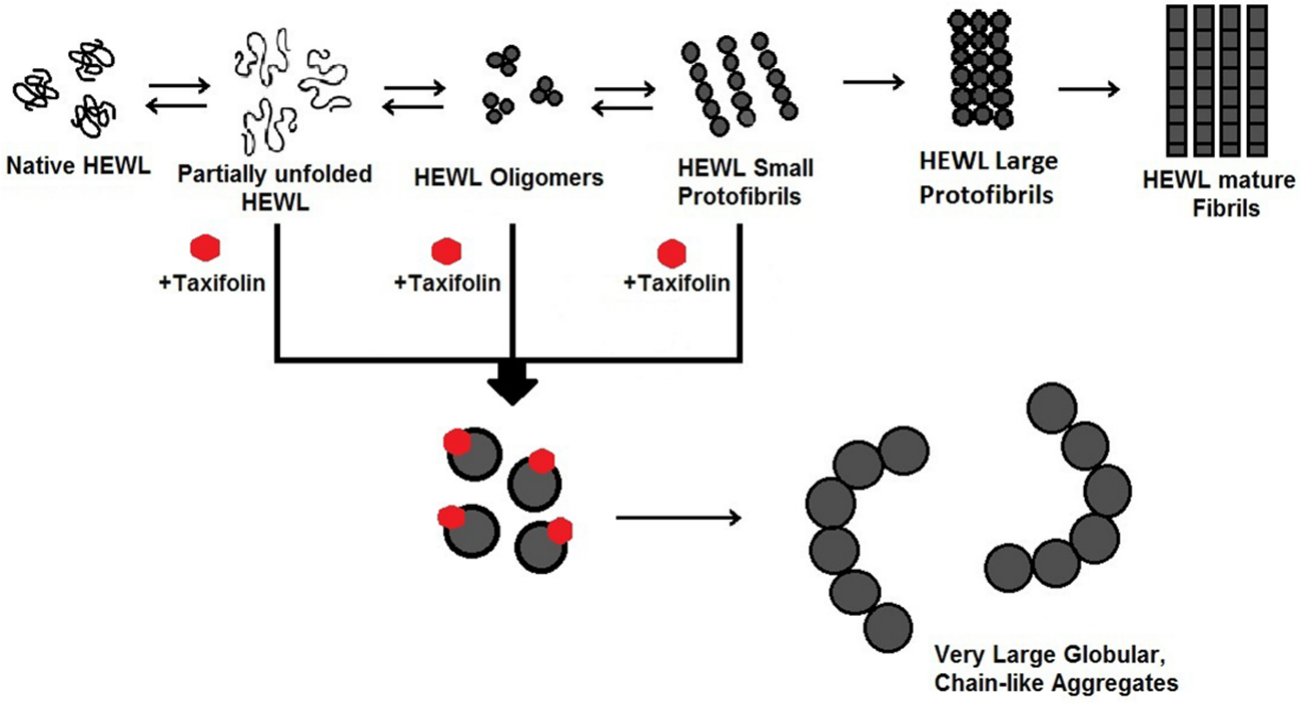
Schematic presentation of a possible mechanism of action on the HEWL amyloid fibril formation. Taxifolin alters the HEWL amyloid assembly pathway yielding very large globular, chain-like aggregates.

In the present study, taxifolin was found to effectively inhibit amyloid fibrillation of HEWL. Our results suggest that it binds to prefibrillar species produced in the course of the aggregation process with the capacity of redirecting the protein fibrillation pathway toward formation of very large globular assemblies, arranged in a chain-like structure (Fig 11). Moreover, cytotoxicity experiments showed that these large assemblies induced by taxifolin are totally nontoxic compared to amyloid fibrils produced in the absence of taxifolin. The results presented may be useful for gaining a deeper insight into possible mechanisms of amyloid fibrillation inhibition by taxifolin and may provide useful guidelines in relation to screening for novel inhibitors.

## Supporting information

S1 File **Figure A. ThT fluorescence intensity of HEWL (50 μM) in the presence of various concentrations of taxifolin (0–200 μM)**. Samples were incubated in 50 mM glycine (pH 2.2) at 57°C for 7 days. **Figure B. AFM images of HEWL (50 μM) incubated alone in 50 mM glycine buffer (pH 2.2) at 57°C for 7 days**. Arrows from left to right indicate worm-like fibrils, rope-like fibrils, and annular structures, respectively. The scale bars represent 500 nm. **Figure C. Effect of taxifolin on the surface hydrophobicity of HEWL**. Protein samples (50 μM) were incubated in 50 mM glycine (pH 2.2) at 57°C either alone (●) or with 25 (○), 50 (♦), or 100 (◊) μM taxifolin for 7 days followed by Nile red fluorescence measurement. The changes in the Nile red fluorescence emission spectrum after treatment with native HEWL is also provided (▲). **Figure D. The influence of total protein concentration on the inhibitory effect of taxifolin on HEWL fibrillation**. This was measured by monitoring the ThT fluorescence emission decrement observed after 7 days of incubation in the presence of 100 μM taxifolin, as compared to that found in its absence. **Table A. Quantification of Congo red binding. HEWL was incubated alone or with various concentrations of taxifolin for 7 days under amyloidogenic conditions**.
(DOCX)
