# Supplementary material for: Inhibition of HEWL fibril formation by taxifolin: Mechanism of action
Source: PLoS One. 2017 Nov 13;12(11):e0187841. doi: 10.1371/journal.pone.0187841 (PMC5683630; doi:10.1371/journal.pone.0187841)
Supplement: S1 File — Figure A. ThT fluorescence intensity of HEWL (50 μM) in the presence of various concentrations of taxifolin (0–200 μM). Samples were incubated in 50 mM glycine (pH 2.2) at 57°C for 7 days. Figure B. AFM images of HEWL (50 μM) incubated alone in 50 mM glycine buffer (pH 2.2) at 57°C for 7 days. Arrows from left to right indicate worm-like fibrils, rope-like fibrils, and annular structures, respectively. The scale bars represent 500 nm. Figure C. Effect of taxifolin on the surface hydrophobicity of HEWL. Protein samples (50 μM) were incubated in 50 mM glycine (pH 2.2) at 57°C either alone (●) or with 25 (○), 50 (♦), or 100 (◊) μM taxifolin for 7 days followed by Nile red fluorescence measurement. The changes in the Nile red fluorescence emission spectrum after treatment with native HEWL is also provided (▲). Figure D. The influence of total protein concentration on the inhibitory effect of taxifolin on HEWL fibrillation. This was measured by monitoring the ThT fluorescence emission decrement observed after 7 days of incubation in the presence of 100 μM taxifolin, as compared to that found in its absence. Table A. Quantification of Congo red binding. HEWL was incubated alone or with various concentrations of taxifolin for 7 days under amyloidogenic conditions. (DOCX) [file pone.0187841.s001.docx]

**Supporting information**

**S1 File Figure A.**

**
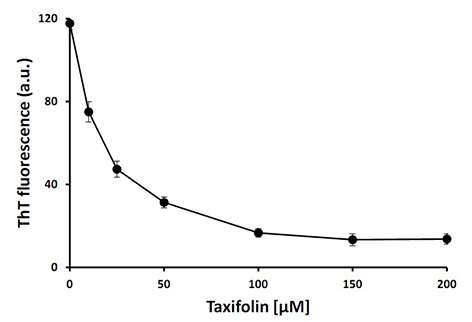
**

**S1 File Figure B.**

**
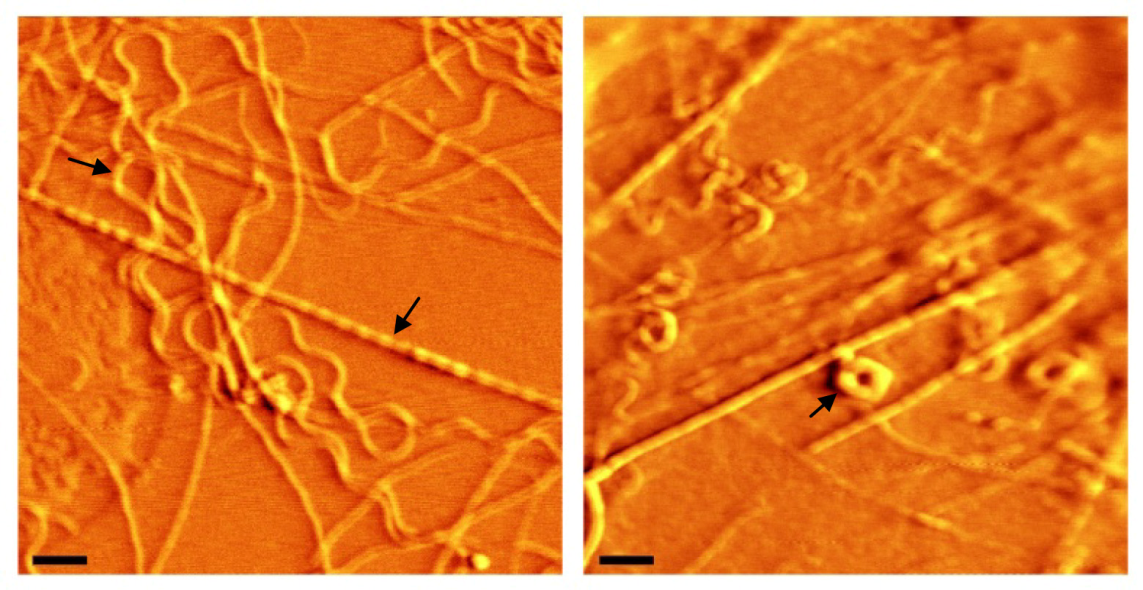
**

**S1 File Figure C.**

**
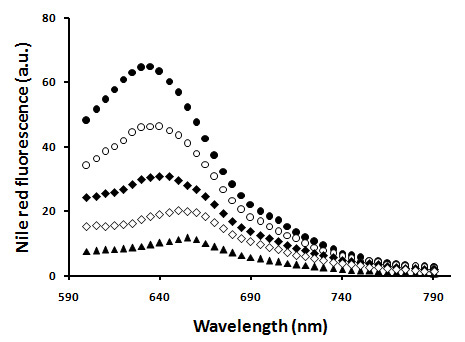
**

**S1 File Figure D.**


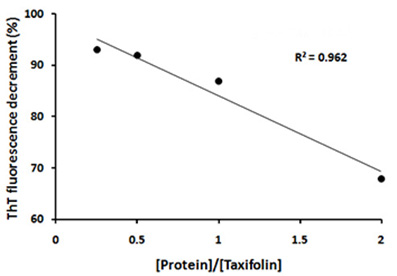


**S1 File Table A.**

|  | Congo red binding |
| --- | --- |
| Fresh HEWL | 0.028 ± 0.001 |
| 0 µM taxifolin | 3.763 ± 0.135 |
| 25 µM taxifolin | 3.604 ± 0.063 |
| 50 µM taxifolin | 2.866 ± 0.139 |
| 100 µM taxifolin | 1.745 ± 0.053 |
